# Transfer of Th17 from Adult Spontaneous Hypertensive Rats Accelerates Development of Hypertension in Juvenile Spontaneous Hypertensive Rats

**DOI:** 10.1155/2021/6633825

**Published:** 2021-02-20

**Authors:** Jee Young Kim, Eunjo Lee, Soohyeon Koo, Cheong-Wun Kim, InKyeom Kim

**Affiliations:** ^1^Department of Pharmacology, School of Medicine, Kyungpook National University, Daegu 41944, Republic of Korea; ^2^Cardiovascular Research Institute, Kyungpook National University, Daegu 41944, Republic of Korea; ^3^BK21 Plus KNU Biomedical Convergence Program, School of Medicine, Kyungpook National University, Daegu 41944, Republic of Korea

## Abstract

Hypertension develops in the recipient rats that are transferred with the activated T helper (Th) 17 cells of the donor rats exposed to high-fructose or high-salt intake. This result suggests that a pathologic Th17 cell plays a role in the development and maintenance of hypertension. Here, we tested the hypothesis that the transfer of Th17 cells from adult spontaneous hypertensive rats (SHR) accelerates the development of hypertension in juvenile SHR. The tail-cuff method was used to measure systolic blood pressure. T cell (Th17 and regulatory T (Treg)) profiling was analyzed by flow cytometry. The expressions of Th17-related interleukin- (IL-) 17A and Treg-related IL-10 were measured by ELISA. Th17 cells isolated from adult SHR were intraperitoneally injected into juvenile recipient SHR and Wistar-Kyoto rats (WKY). SHR exhibited prominent development of hypertension at 15 weeks. The proportion of CD4^+^IL-17A^+^ (Th17) cells among Th cells increased whereas the proportion of CD4^+^FoxP3^+^ (Treg) cells decreased in SHR, as compared to WKY. The serum levels of IL-17A increased gradually with aging in SHR, but the serum levels of IL-10 did not. The serum levels of IL-17A and IL-10 seemed to be well related to the proportion of Th17 cells and Treg cells, respectively. Injection of Th17 cells isolated from adult SHR accelerates the development of hypertension in juvenile SHR but not in juvenile WKY though it increased the proportion of Th17 cells in juvenile recipient WKY and SHR. The transfer of Th17 cells from adult SHR accelerates the development of hypertension in juvenile SHR. These results implicate that the hypertension in SHR is ascribed to activation of Th17 cells.

## 1. Introduction

Hypertension develops in a complex phenomenon of the sympathetic nervous system and the renin-angiotensin-aldosterone system. It is difficult to restore healthy blood pressure from hypertension by the limitation of the sympathetic nervous system and renin-angiotensin-aldosterone system. Recently, clinical and experimental researches suggest a contribution of immune mechanisms to the development of hypertension [1]. The lack of B and T cells by recombination activating gene-1-knockout (Rag1^−/−^) mice plays a role in inhibiting immune response and cytokine signaling in high-salt or angiotensin II-induced hypertension [2]. Interleukin- (IL-) 17^−/−^ knockout results in blunted hypertension and reduced renal or vascular dysfunction. Neutralizing antibodies against IL-17A, IL-17F, or IL-17RA receptor subunits have shown the potential to lower blood pressure and improve hypertension-related end-organ damage [3, 4]. Therefore, activation of immune cells in hypertension models is expected to promote the inflammatory response and increase blood pressure [5].

The SHR strain is widely used to model human hypertension, metabolic syndrome, and many other pathophysiological conditions [6, 7]. The development of the SHR strain is by selective breeding of the WKY stock for hypertension. In hypertension in SHR, which gradually progresses worse with aging, the stage can be divided into the prehypertension phase (0-4 weeks), labile phase (5-10 weeks), and established phase (>3 months) [8]. The blood pressure in SHR is normal in the prehypertensive stage, whereas it increases with aging to become hypertension in an established stage [9].

Th17 cells are proinflammatory cells that secrete proinflammatory cytokines to activate the immune response, promote high blood pressure, and accelerate the damage to target organs [10–12]. We previously reported that Th17 cells in Dahl salt-sensitive rats (SS) with high-fructose intake become pathogenic Th17 cells, consequently resulting in increased blood pressure as well as increased proinflammatory cytokine activity. Moreover, transfer of the pathogenic Th17 cells increases Th17 cell proportion and blood pressure in recipient SS [13]. Cytokines such as TGF-*β*, IL-6, and IL-1*β* activate ROR*γ*, a transcription factor, by which they play an important role in the differentiation from naive CD4^+^ T cells to the Th17 phenotype [14, 15]. In addition, IL-23 activates the Th17 transcription factors STAT3, ROR*α*, and ROR*γ*t to maintain the Th17 phenotype and long-term proinflammatory signature [16, 17]. The normal immune system is well regulated to prevent and defend against shifting toward proinflammatory response. Treg cells are immunosuppressive and generally inhibit or downregulate the proinflammatory responses. The Treg cells are identified by the expression of IL-2R*α* (CD25) as a functional marker and by the upregulation of FoxP3 as a master transcriptional factor [18, 19]. The balance between the Th17 cells and Treg cells is a key factor in maintaining immune homeostasis and health. When the Treg cell proportion decreases or a dysfunction occurs, the immune system shifts toward a proinflammatory response [20].

In SHR, Treg cells in the peripheral blood mononuclear cells (PBMCs) and in the spleen are downregulated and Th17 cells are upregulated, compared with those in the WKY [21, 22]. Thus, this immune imbalance between the Th17 cell and Treg cell in SHR is an important mechanism triggering a proinflammatory response and inducing high blood pressure [1, 20, 21]. In the labile phase of both WKY and SHR, the proportion of Th17 cells and Treg cells is not unchanged. In the established phase, SHR shows that the proportion of Th17 cells increases and the proportion of Treg cells decreases, but WKY shows that the proportion of Th17 cells remains unchanged and the proportion of Treg cells increases.

This competitive relationship between the Th17 cell and Treg cell is maintained in either physiological or pathological condition. However, the preference toward proinflammatory response by Th17 cell dominance in the immune system presides in SHR, by which hypertension may develop. According to this knowledge, we hypothesize that the transfer of Th17 cells from adult SHR accelerates the alteration of the immune system and the development of hypertension in juvenile SHR.

## 2. Materials and Methods

### 2.1. Animals

The investigation was conducted in accordance with the National Institutes of Health Guide for the Care and Use of Laboratory Animals and was approved by the Institutional Review Board of Kyungpook National University (approval no. 2019-0074). Every effort was made to minimize both the number of animals used and their suffering. Male juvenile (5-week-old) and adult (15-week-old) WKY and SHR were purchased from SLC, Inc., Japan.

### 2.2. Blood Pressure Measurements

The systolic blood pressure (SBP) of the rats was measured weekly by the tail-cuff method for thirteen weeks. Rats were warmed for 10 min at 35°C and then placed in plastic restrainers. The rat tail was inserted into a cuff with a pneumatic pulse sensor. Blood pressure values were recorded on a CODA system (Kent Scientific Corporation, Torrington, CT, USA) and were averaged from at least 10 consecutive readings obtained from each rat.

### 2.3. Serum Level of Cytokines

Under anesthesia, serums were collected from WKY and SHR and stored at -80°C for analysis. Serum levels of IL-17A and IL-10 were analyzed by using enzyme-linked immunosorbent assay (ELISA) kits: IL-17A (BMS635; Thermo Fisher Scientific, Waltham, MA, USA) and IL-10 (R1000; R&D Systems, Inc., Minneapolis, MN, USA). The optical density value was measured at 450 nm, and the concentrations of IL-17A and IL-10 were calculated with the standard curves.

### 2.4. Flow Cytometry

The single-cell suspension method of PBMCs was described in our previous study [13]. The single-cell suspension method of the spleen and kidney was described in previous studies [23, 24]. Rats were anesthetized with sodium pentobarbital (50 mg/kg intraperitoneally) for sacrifice, and then, the serum, spleen, and kidney were taken and stored at -80°C in liquid nitrogen until further studies. Monoclonal antibodies FITC mouse anti-rat CD3 (1 : 100, clone G4.18, #554832, BD Bioscience, Franklin Lakes, NJ, USA) and PE mouse anti-rat CD4 (1 : 100, clone OX-35, #554838, BD Bioscience) as T cell surface markers stained the PBMCs, spleen, and kidney from WKY and SHR. CD3 and CD4 were used to identify T helper (Th) cells. To identify Th17 cells or Treg cells, the stained CD3 and CD4 cells were washed and fixed and then were permeabilized using fix/perm concentrate (BD Bioscience). The cells were incubated with the monoclonal antibody APC rat anti-rat IL-17A (1 : 100, clone eBio17B7, #17-7177-81, Thermo Fisher Scientific) for Th17 cells or PerCP-Cyanine5.5 rat anti-rat FoxP3 (1 : 100, clone FJK-16s, #14-5773-82, Thermo Fisher Scientific) for Treg cells. After incubation, the cells were washed and run through a four-color flow cytometer (FACSCalibur; BD Biosciences). Data were collected using CellQuest.

### 2.5. Isolation of Th17 Cells and Adoptive Cell Transfer

In peripheral blood obtained from adult SHR as a donor, PBMCs were separated by Ficoll-Paque PLUS gradient centrifugation (GE Healthcare, Chicago, IL, USA). Th17 cells were sorted by high-speed flow cytometry (FACSAria; BD Biosciences) to a purity of >90% as verified by postsort analysis using CD3-FITC (BD Biosciences), CD4-PE (BD Bioscience), and IL-17A-APC (1 : 100, Thermo Fisher Scientific). For an isolation strategy of Th17 cells from adult SHR, lymphocytes were selected based on forward scatter (FSC) and side scatter (SSC, R1) and then were gated with the following parameters: CD3^+^CD4^+^ (R2). Th17 cells were isolated by using CD3^+^CD4^+^IL-17A^+^ (R3). The sorting strategy for Th17 cells from adult SHR is shown in Supplementary Figure 3. Before adoptive cell transferring, SBP of adult or juvenile recipient rats was measured by the tail-cuff method. The recipient rats received 300 *μ*L of 1 × 10^5^ of adoptive Th or Th17 cells through intraperitoneal injections (using a 1.0 mL syringe with a 26-gauge needle). After transfer of adoptive Th or Th17 cells into the adult or juvenile recipient rats, the recipient rats were allowed to stabilize for one day, and then, SBP of the recipient rats was measured every day for 3 consecutive days. At the fourth day of the experiment, the recipient rats were anesthetized for sacrifice with sodium pentobarbital (50 mg/kg intraperitoneally). The serum, spleen, and kidney were collected and stored at -80°C until further studies. Single-cell suspensions of the PBMCs, spleen, and kidney were measured by using the four-color flow cytometer. The representative flow cytometry gating strategy is shown in Supplementary Figure 5a-c.

### 2.6. Statistics

Data were presented as mean ± standard error of mean (SEM) and analyzed using Prism (Graphpad Software). Comparison of means between two groups was analyzed by Student's *t*-tests. Analysis of variance (ANOVA) followed by Duncan post hoc tests was applied to compare multiple groups. A *P* value < 0.05 was considered statistically significant.

## 3. Results

### 3.1. The Systolic Blood Pressure and Body Weight in WKY and SHR

**Figure 1:**
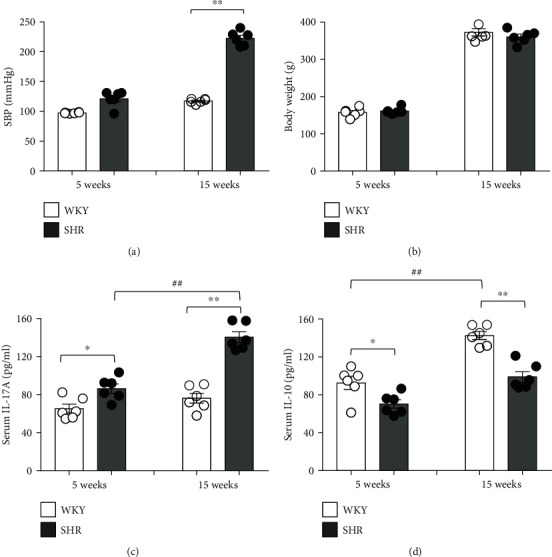
The systolic blood pressure (SBP), body weight, and serum levels of cytokines in Wistar-Kyoto rats (WKY) and spontaneous hypertensive rats (SHR). (a) SBP was measured using the tail-cuff method. SBP of SHR significantly higher at adult (15 weeks) compared with juvenile (5 weeks). (b) The body weights of the WKY and SHR revealed comparable in 5 weeks and 15 weeks. The serum levels of (c) a proinflammatory cytokine (IL-17A) and (d) an anti-inflammatory cytokine (IL-10) were measured using ELISA in juvenile and adult rats. The serum levels of IL-17A were higher in SHR than in WKY, whereas the levels of IL-10 were lower in SHR than in WKY. Data are the mean ± SEM of 6 independent experiments. Most SEMs were too small to be seen out of symbols (^∗^*P* < 0.05, ^∗∗^*P* < 0.01 vs. WKY; ^##^*P* < 0.01 vs. corresponding juvenile rats by two-way ANOVA followed by the post hoc Duncan test).

The SBP of 5-week-old WKY and SHR were comparable, but SHR at 15 weeks showed significantly higher SBP than WKY (Figure 1(a), *n* = 6). In weekly SBP measurement of 4- to 16-week-old WKY and SHR for 13 weeks, the SBP of SHR began to increase significantly at 6 weeks, so early-stage hypertension developed in SHR. More severe hypertension developed in 10-week-old SHR (*P* < 0.01), whereas normal SBP were maintained in WKY (Supplementary Figure 1a). However, body weight was not different between 15-week-old WKY and SHR (Figure 1(b), Supplementary Figure 1b).

### 3.2. Serum Levels of Cytokines in WKY and SHR

To explain different blood pressures between WKY and SHR, we measured the serum levels of IL-17A (a proinflammatory cytokine) and IL-10 (an anti-inflammatory cytokine) using an ELISA kit in juvenile (5 weeks old) and adult groups (15 weeks old). The serum levels of IL-17A were already higher in juvenile SHR than in juvenile WKY (*P* < 0.05), much higher in adult SHR than in adult WKY (*P* < 0.01), and also significantly higher in adult SHR than in juvenile SHR (*P* < 0.01). However, no significant difference was found in the serum levels of IL-17 between adult and juvenile WKY (Figure 1(c)). On the contrary, the serum levels of IL-10 were higher in juvenile WKY than in juvenile SHR (*P* < 0.05), much higher in adult WKY than in adult SHR (*P* < 0.01), and also significantly higher in adult WKY than in juvenile WKY (*P* < 0.01; Figure 1(d)).

### 3.3. T Cell Profiles of the PBMCs and Spleen in WKY and SHR

**Figure 2:**
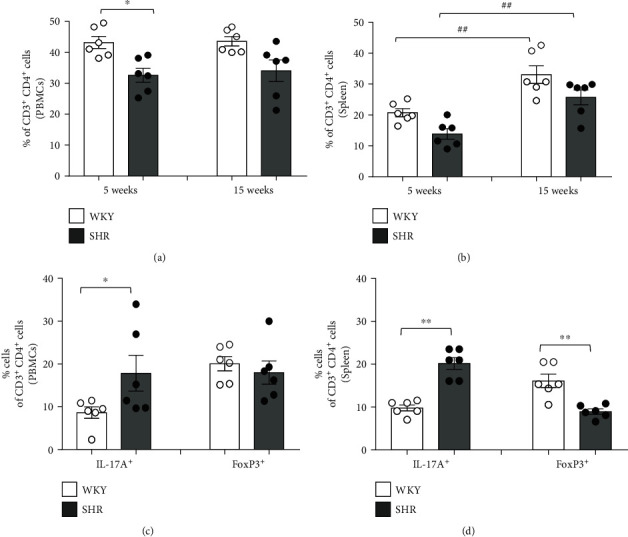
T cell profiles of the peripheral blood mononuclear cells (PBMCs) and spleen in WKY and SHR. Quantification of flow cytometric analysis revealed the proportion of CD3^+^CD4^+^ (Th) cells of the (a) PBMCs and (b) spleen in juvenile and adult rats. The proportion of CD3^+^CD4^+^ (Th) cells of PBMCs was higher in WKY than in SHR. The proportion of CD3^+^CD4^+^ (Th) cells of the spleen revealed comparable in 5 weeks and 15 weeks, but increased at 15 weeks compared with 5 weeks (^∗^*P* < 0.05, ^∗∗^*P* < 0.01 vs. WKY; ^##^*P* < 0.01 vs. corresponding juvenile rats by two-way ANOVA followed by the post hoc Duncan test). Quantification of flow cytometric analysis revealed the proportion of CD4^+^IL-17A^+^ (Th17) and CD4^+^FoxP3^+^ (Treg) cells among CD3^+^CD4^+^ (Th) cells of the (c) PBMCs and (d) spleen in adult WKY and SHR by the gating strategy of flow cytometry. There was a higher proportion of CD4^+^IL-17A^+^ (Th17) cells of PBMCs in SHR than in WKY, and there was a lower proportion of CD4^+^FoxP3^+^ (Treg) cells in SHR than in WKY. Data are the mean ± SEM of 6 independent experiments (^∗^*P* < 0.05 vs. WKY by Student's *t*-test).

To evaluate the different observations of the immune responses between SHR and WKY, T lymphocyte profiling among the PBMCs and spleen was analyzed using flow cytometry. In PBMCs, the proportion of CD3^+^CD4^+^ (T helper (Th)) cells was analyzed in 5-week-old and 15-week-old WKY and SHR, and significant difference was found in the proportion of CD3^+^CD4^+^ (Th) cells in 5-week-old SHR compared to WKY (*P* < 0.05), whereas 15-week-old rats were not (Figure 2(a)). Although Th cells of PBMCs had no significant difference between 15-week-old WKY and SHR, the proportion of CD4^+^IL-17A^+^ (Th17) cells among Th cells was revealed to be much more in SHR than in WKY (*P* < 0.05). However, there was no significant difference in the proportion of CD4^+^FoxP3^+^ (Treg) cells of PBMCs between WKY and SHR (Figure 2(c)).

In the spleen, the proportion of CD3^+^CD4^+^ (Th) cells between WKY and SHR has no statistically significant difference but was increased in 15-week-old rats compared with 5-week-old rats (*P* < 0.01, Figure 2(b)). The proportion of Th17 cells was revealed to be much higher in SHR than in WKY (*P* < 0.01), and the proportion of Treg cells of the spleen was also revealed to be lower in SHR than in WKY (*P* < 0.01; Figure 2(d)).

According to these results, the serum levels of IL-17A and IL-10 seemed to be well related to the proportion of Th17 cells and Treg cells, respectively, among the PBMCs and spleen.

### 3.4. Effect of Transferring Th17 Cells from Adult SHR on the Blood Pressure of Juvenile WKY and SHR

**Figure 3:**
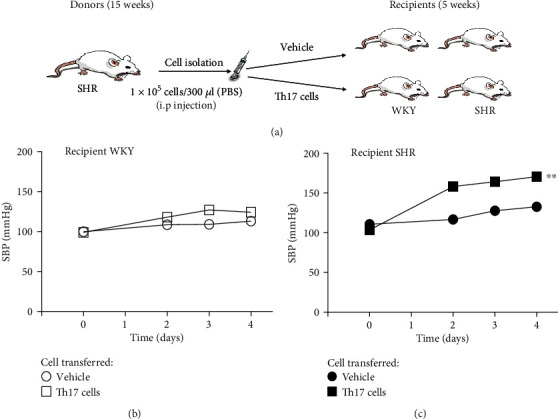
Accelerated development of hypertension in juvenile SHR by transfer of adult Th17 cells. (a) Schematic illustration shows that Th17 cells from adult donor SHR or the vehicle was transferred into juvenile recipient WKY or SHR. Intraperitoneal injections with Th17 cells from adult SHR or the vehicle were administrated into juvenile recipient WKY or SHR. SBP was measured in juvenile recipient (b) WKY and (c) SHR for 4 days using the tail-cuff method. The transfer of Th17 cells from adult SHR into juvenile recipient SHR significantly increased SBP than the vehicle group, which was not seen in juvenile recipient WKY. Data are the mean ± SEM of 6 independent experiments (^∗∗^*P* < 0.01 vs. vehicle group by repeated measures ANOVA).

To explore whether the increased proportion of Th17 cells in blood contributes to increased SBP in juvenile rats, the Th17 cells were isolated from adult donor SHR and then transferred into the juvenile recipient WKY or SHR through intraperitoneal injections (Figure 3(a)). Before intraperitoneal injection with the isolated Th17 cells from adult SHR, SBP of the juvenile recipient WKY and SHR was measured at 0 day, which was virtually within normal range. SBP of the juvenile recipient WKY by transferring Th17 cells was moderately increased but did not reach to hypertension (Figure 3(b)). When the Th17 cells from adult SHR were also transferred into adult recipient 15-week-old WKY, SBP of the adult recipient WKY was insignificantly increased at day 2 but was decreasing at day 3 and went back within the normal SBP at day 4 (Supplementary Figure 2b). Therefore, transferring Th17 cells from adult SHR to adult WKY or juvenile WKY did not affect their SBP. However, SBP of the juvenile recipient SHR by transferring Th17 cells from adult SHR was accelerated and maintained hypertension (Figure 3(c)). Therefore, the Th17 cells from adult SHR accelerated SBP and reached hypertension in only juvenile recipient SHR but not both juvenile and adult recipient WKY. When Th cells of adult SHR were transferred, there was no significant increase in blood pressure in both juvenile recipient WKY and SHR (Supplementary Figure 4a).

### 3.5. T Cell Profiles of the Juvenile Recipient WKY and SHR in the PBMCs, Spleen, and Kidney after Transferring Th17 Cells of Adult SHR

In the previous results of SBP measurement, transferring Th17 cells from adult donor SHR accelerated the SBP of juvenile recipient SHR but not both juvenile and adult WKY (*n* = 4-6). The proportion of Th17 cells or Treg cells of the PBMCs, spleen, and kidney in juvenile recipient WKY and SHR needed to be profiled by flow cytometry with staining of CD4^+^IL-17A^+^ for Th17 cells or CD4^+^FoxP3^+^ for Treg cells.

**Figure 4:**
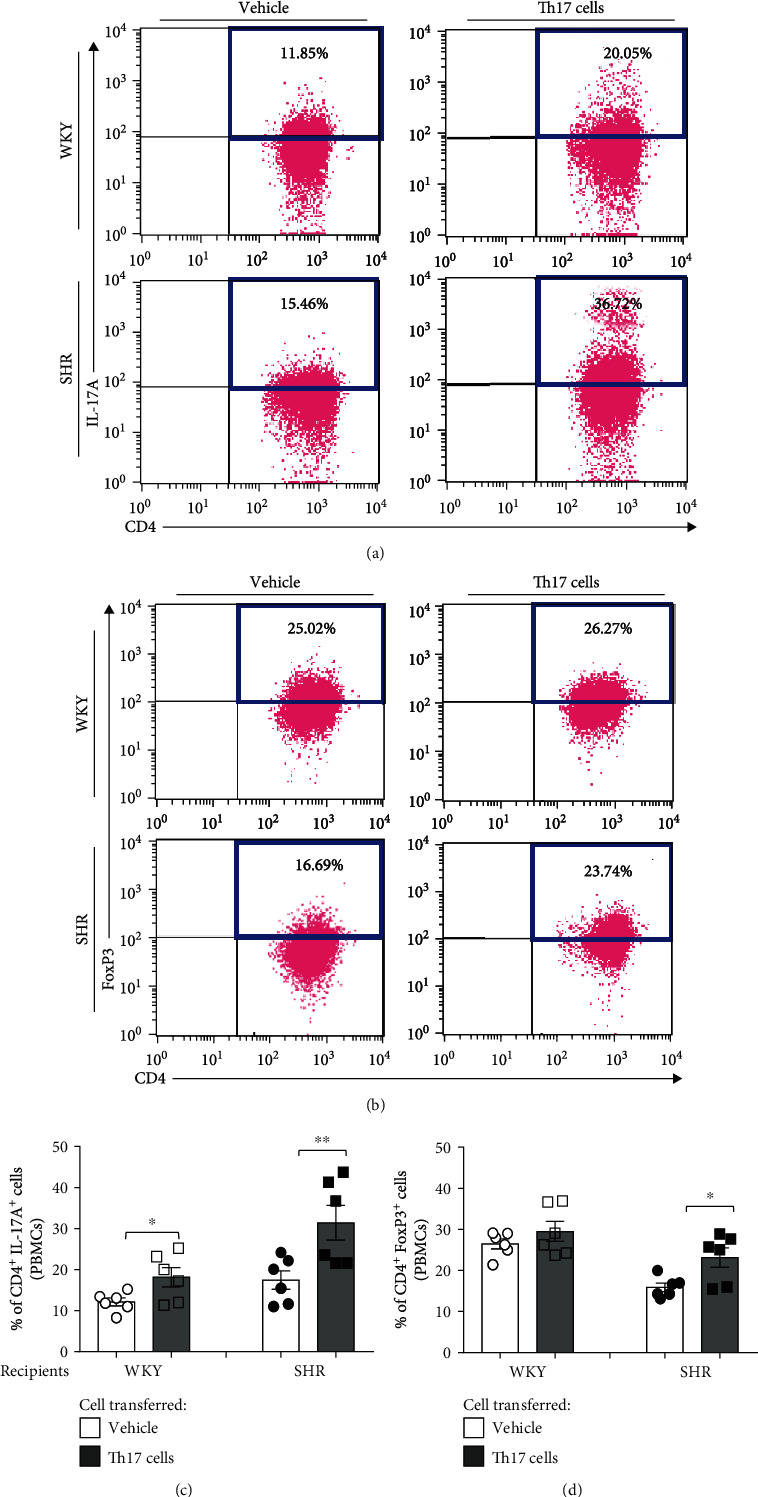
T cell profiles of PBMCs in juvenile recipient WKY and SHR for 4 days after transferring Th17 cells from adult donor SHR. Representative flow cytometric analysis revealed the proportion of (a) CD4^+^IL-17A^+^ (Th17) cells and (b) CD4^+^FoxP3^+^ (Treg) cells of PBMCs in juvenile recipient WKY or SHR. (c) Quantification revealed that CD4^+^IL-17A^+^ (Th17) cells after the transfer of Th17 cells significantly increased compared with the vehicle in juvenile recipient WKY and SHR. (d) Quantification revealed that the proportion of CD4^+^FoxP3^+^ (Treg) cells after transfer of Th17 cells significantly increased compared with the vehicle in juvenile recipient SHR. Data are the mean ± SEM of 6 independent experiments (^∗^*P* < 0.05, ^∗∗^*P* < 0.01 vs. vehicle group by two-way ANOVA followed by the post hoc Duncan test).

In flow cytometry for PBMCs, the proportion of Th17 cells in the juvenile recipient WKY or SHR was significantly increased by transferring Th17 cells from adult SHR compared with transferring the vehicle (*P* < 0.05 in WKY and *P* < 0.01 in SHR; Figures 4(a) and 4(c)). When Th cells of adult SHR are transferred, there was significant increase in the proportion of Th17 cells in juvenile recipient SHR (*P* < 0.05, Supplementary Figure 4b). By transferring Th17 cells of adult SHR, the proportion of Treg cells was significantly increased in the juvenile recipient SHR (*P* < 0.05) but not in the juvenile recipient WKY (Figures 4(b) and 4(d)).

**Figure 5:**
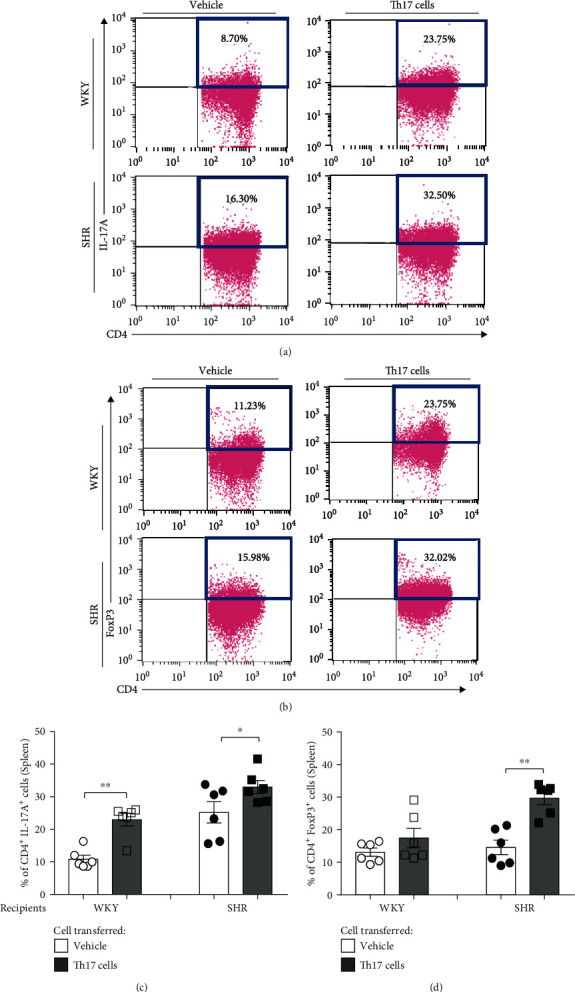
T cell profiles of the spleen in juvenile recipient WKY and SHR for 4 days after transferring Th17 cells from adult donor SHR. Representative flow cytometric analysis revealed the proportion of (a) CD4^+^IL-17A^+^ (Th17) cells and (b) CD4^+^FoxP3^+^ (Treg) cells of the spleen in juvenile recipient WKY or SHR. (c) Quantification revealed that the proportion of CD4^+^IL-17A^+^ (Th17) cells after transfer of Th17 cells significantly increased compared with the vehicle in juvenile recipient WKY and SHR. (d) Quantification revealed that the proportion of CD4^+^FoxP3^+^ (Treg) cells after transfer of Th17 cells significantly increased compared with the vehicle in juvenile recipient SHR. Data are the mean ± SEM of 6 independent experiments (^∗^*P* < 0.05, ^∗∗^*P* < 0.01 vs. vehicle group by two-way ANOVA followed by the post hoc Duncan test).

In flow cytometry for the spleen, transferring Th17 cells from adult SHR induced the increase in the proportion of Th17 cells in the juvenile recipient WKY and SHR compared with transferring the vehicle (*P* < 0.01 in WKY and *P* < 0.05 in SHR; Figures 5(a) and 5(c)). When Th cells of adult SHR are transferred, there was significant increase in the proportion of Th17 cells in juvenile recipient WKY (*P* < 0.05, Supplementary Figure 4c). Moreover, transferring Th17 cells from adult SHR did not change the proportion of spleen Treg cells in the juvenile recipient WKY but induced the increase in the proportion of spleen Treg cells in the juvenile recipient SHR (*P* < 0.01) compared with transferring the vehicle (Figures 5(b) and 5(d)).

**Figure 6:**
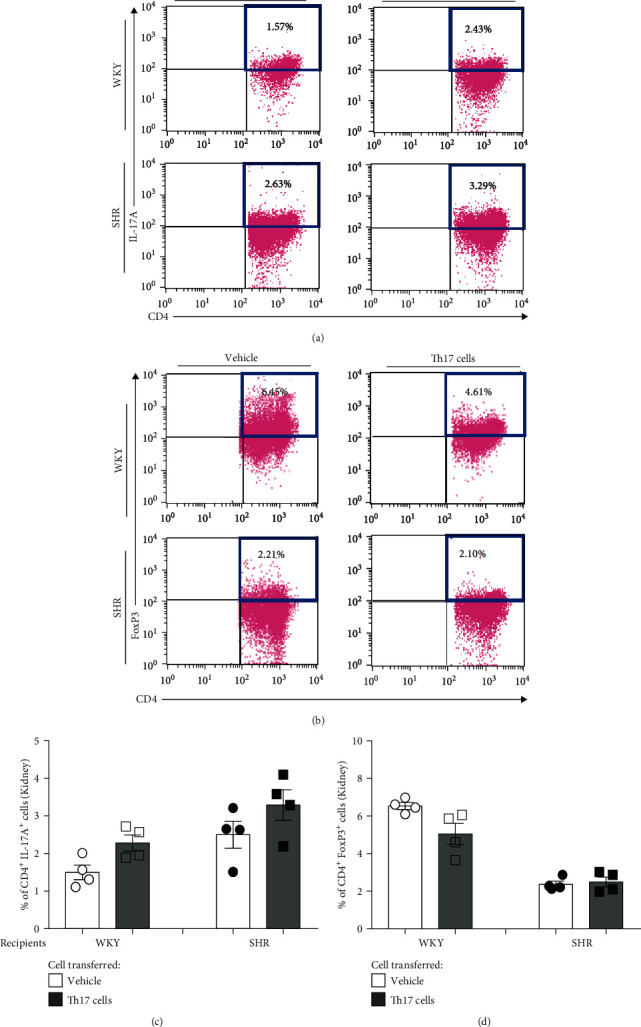
T cell profiles of the kidney in juvenile recipient WKY and SHR for 4 days after transferring Th17 cells from adult donor SHR. Representative flow cytometric analysis revealed the proportion of (a) CD4^+^IL-17A^+^ (Th17) cells and (b) CD4^+^FoxP3^+^ (Treg) cells of kidney lymphocytes in juvenile recipient WKY or SHR. Quantification revealed that the proportion of (c) CD4^+^IL-17A^+^ (Th17) cells and (d) CD4^+^FoxP3^+^ (Treg) cells after transfer of Th17 cells was comparable with transferring the vehicle in juvenile recipient WKY and SHR. Data are the mean ± SEM of 4 independent experiments.

In flow cytometry for the kidney, the proportion of renal Th17 cells in both juvenile recipient WKY and SHR after transferring Th or Th17 cells from adult SHR or the vehicle was insignificantly changed by transferring Th17 cells from adult SHR compared with the vehicle (Figures 6(a) and 6(c), Supplementary Figure 4d). Similarly, the proportion of renal Treg cells in both juvenile recipient WKY and juvenile SHR was also insignificantly changed by transferring Th17 cells of adult SHR compared with the vehicle (Figures 6(b) and 6(d)).

## 4. Discussion

This study demonstrates that the transfer of adult Th17 cells accelerates the development of hypertension in juvenile SHR. The transfer of Th17 cells isolated from adult donor SHR blood significantly increases the proportion of CD4^+^IL-17A^+^ (Th17) cells in the PBMCs and the spleen of recipient juvenile rats and induces early hypertension in the juvenile recipient SHR. The transfer of Th17 cells accelerates the occurrence of hypertension in the juvenile recipient SHR but not in both adult and juvenile recipient WKY. These findings suggest that Th17 cells from adult SHR play a role in the development of hypertension and that juvenile recipient SHR may be more susceptible to an increase in the proportion of CD4^+^IL-17A^+^ (Th17) cells than the juvenile recipient WKY.

In addition, this study reveals that the proportion of CD4^+^IL-17A^+^ (Th17) cells among CD3^+^CD4^+^ (Th) cells in the PBMCs and the spleen significantly increases in both recipient WKY and SHR, whereas the proportion of CD4^+^FoxP3^+^ (Treg) cells increases in the recipient SHR but not in recipient WKY. These findings imply that the increased proportion of CD4^+^FoxP3^+^ (Treg) cells in the PBMCs and the spleen is insufficient to offset the effect of the increased proportion of CD4^+^IL-17A^+^ (Th17) in the recipient SHR. Despite the absence of an increase in the proportion of CD4^+^FoxP3^+^ (Treg) cells in the recipient WKY, the increased proportion of CD4^+^IL-17A^+^ (Th17) cells did not affect the onset of hypertension. Therefore, the recipient SHR may be more susceptible to an increase in the proportion of CD4^+^IL-17A^+^ (Th17) cells than the WKY.

Several studies on early programmed hypertension showed the importance of T cell activation. Activated T cells and T cell-driven cytokines cause vasoconstriction and vascular remodeling and drive proinflammatory responses that ultimately promote the development of hypertension [25]. IL-17A that is mostly produced by activated Th17 cells is a key factor for proinflammatory responses that induce systemic endothelial dysfunction, vascular oxidative stress, and arterial hypertension [12, 13]. Blood pressure is not different between WKY and SHR at 3 to 5 weeks, whereas the levels of IL-17A are higher in SHR and the levels of IL-10 are higher in WKY [9, 21, 26]. Therefore, early elevated levels of the proinflammatory IL-17A produced by Th17 cells are important in the development of spontaneous hypertension in SHR. In this experiment, the transfer of Th17 cells enhances the proportion of CD4^+^IL-17A^+^ (Th17) cells in the PBMCs and the spleen of recipient SHR by which hypertension consequently develops. According to this experiment, Th17 cell activation involves the development of hypertension in the recipient SHR but not in WKY. This is probably caused by an instinctively maintained immune balance in WKY.

Besides, 15-week-old SHR exhibits the higher proportion of CD4^+^IL-17A^+^ (Th17) cells in the PBMCs and the spleen and the lower proportion of CD4^+^FoxP3^+^ (Treg) cells in the spleen than 15-week-old WKY does. Because the CD4^+^FoxP3^+^ (Treg) cells secreting IL-10 prevent the development of hypertension contrary to Th17 cells, both changes in the high serum levels of IL-17A and low serum levels of IL-10 in both juvenile and adult SHR promote proinflammatory responses, reduce anti-inflammatory responses, and play a critical role in the development of hypertension. Another study also reported that the different proportions of Treg cells between WKY and spontaneously hypertensive stroke-prone rats (SHRSP) play a crucial role in the development of hypertension. The proportion of Treg cells in WKY is similar in young (3 weeks) and old (15 weeks) ages, whereas it decreases in old SHRSP compared with those of a young age [25]. Treg cells that possess immunosuppressive capabilities in WKY are another key modulator to ameliorate inflammation and blood pressure increment [27, 28]. However, our study shows that the proinflammatory and hypertensive effects of the increased proportion of CD4^+^IL-17A^+^ (Th17) cells in juvenile recipient SHR surpass the anti-inflammatory suppressive response of the increased proportion of CD4^+^FoxP3^+^ (Treg) cells. In contrast, the immune defense mechanisms of the recipient WKY are able to offset the proinflammatory and hypertensive effect of increased proportion of CD4^+^IL-17A^+^ (Th17) cells, and the proportion of CD4^+^FoxP3^+^ (Treg) cells does not even increase. Interestingly, adult SHR has higher proportion of CD4^+^IL-17A^+^ (Th17) cells and lesser proportion of CD4^+^FoxP3^+^ (Treg) cells than adult WKY has. The changes of the proportion of both CD4^+^IL-17A^+^ (Th17) and CD4^+^FoxP3^+^ (Treg) cells increase after the transfer of Th17 cells in recipient SHR, which may be caused by an alteration of immune balance toward proinflammatory response that may be more intensified with the aging process.

The proportion of T cells is slightly different between the PBMCs and the spleen. As the spleen is the largest lymphoid organ that plays a fundamental role in the maintenance of immune homeostasis, the proportion of T cells in the spleen may reflect the overall variation in their proportion in the circulation [26, 29].

## 5. Conclusion

This study demonstrates that the transfer of adult Th17 cells accelerates the development of hypertension in juvenile SHR. The transfer of Th17 cells increases the proportion of CD4^+^IL-17A^+^ (Th17) cells in both juvenile recipient SHR and WKY, but they exhibit different SBP. This is probably due to the involvement of different immune responses by Th17 cell dominance. Also, the higher serum levels of proinflammatory IL-17A and the lower serum levels of anti-inflammatory IL-10 are in the juvenile SHR, compared to WKY. Therefore, the Th17 cell is a key actor in the development of hypertension, so that the Th17 cell may deserve to be an important target to improve hypertensive damage.

## Data Availability

The data supporting the conclusions of this article are available from the corresponding author on reasonable request.

## Supplementary Materials

Supplementary Materials Supplementary Figure 1: the systolic blood pressure (SBP), body weight, and representative flow cytometric analysis revealed the proportion of CD4^+^IL-17A^+^ (Th17) and CD4^+^FoxP3^+^ (Treg) cells by the gating strategy of flow cytometry in WKY and SHR. (a) SBP was measured using the tail-cuff method. SBP of SHR significantly increased by aging compared with that of WKY. (b) The body weights of the WKY and SHR had been monitored weekly for 13 weeks from 4 to 16 weeks. Data are the mean ± SEM of 6 independent experiments. Most SEMs were too small to be seen out of symbols (^∗∗^*P* < 0.01 vs. WKY by repeated measures ANOVA). Representative flow cytometric analysis revealed the proportion of CD4^+^IL-17A^+^ (Th17) cells and CD4^+^FoxP3^+^ (Treg) cells of the (c) PBMCs and (d) spleen in adult WKY or SHR. Supplementary Figure 2: protection of hypertension development in adult WKY from transfer of Th17 cells of adult SHR. (a) Schematic illustration shows that Th17 cells from adult donor WKY or SHR or the vehicle were transferred into adult recipient WKY. (b) SBP was measured in adult recipient WKY for 4 days using the tail-cuff method. SBP was not significantly increased in adult recipient WKY when either vehicle or transfer of Th17 cells from adult donor WKY or SHR was used. Data are the mean ± SEM of 4 independent experiments. Supplementary Figure 3: isolation strategy of Th17 cells from PBMCs of adult SHR. Single-cell suspension of PBMCs from adult SHR was analyzed based on forward scatter (FSC) and side scatter (SSC). Th cells were gated based on the expression of CD3^+^CD4^+^ and then further gated to Th17 cells by the expression of IL-17A. Supplementary Figure 4: SBP measurement and T cell profiles of juvenile recipient WKY and SHR in the PBMCs, spleen, and kidney for 4 days after transferring Th cells or Th17 cells from adult donor SHR. In the recipient WKY group, (a) there was no significant change in blood pressure when either vehicle or Th cells or Th17 cells of adult SHR were transferred. (b) The proportion of Th17 cells was significantly increased in PBMCs when Th17 cells of adult SHR were transferred. (c) The proportion of Th17 cells was significant in the spleen when Th or Th17 cells of adult SHR were transferred. (d) There was no increase in the proportion of the Th17 cell in the kidney. There was no significant difference in the Treg proportion in PBMCs, spleen, and kidney. In the recipient SHR group, (a) there was significant change in blood pressure when Th17 cells of adult SHR were transferred. (b) The proportion of Th17 cells was significantly increased in PBMCs when Th or Th17 cells of adult SHR were transferred. (c) The proportion of Th17 cells was significant in the spleen when Th17 cells of adult SHR were transferred. (d) There was no increase in the proportion of the Th17 cell in the kidney. There was significant difference in the Treg proportion in the PBMCs and spleen when Th17 cells of adult SHR were transferred. Data are the mean ± SEM of 4-6 independent experiments (^∗^*P* < 0.05, ^∗∗^*P* < 0.01 vs. vehicle group by two-way ANOVA followed by the post hoc Duncan test). Supplementary Figure 5a-c: flow cytometric gating strategy for the Th17 and Treg cells from adult SHR. Lymphocytes of the (a) PBMCs, (b) spleen, and (c) kidney were selected based on FSC and SSC and then gated based on the expression of CD3^+^ and CD4^+^. Cells expressing CD3^+^CD4^+^ were further gated to Th17 cells by the expression of CD4^+^IL-17A^+^ or to Treg cells by the expression of CD4^+^FoxP3^+^.
