# Supplementary material for: Transfer of Th17 from Adult Spontaneous Hypertensive Rats Accelerates Development of Hypertension in Juvenile Spontaneous Hypertensive Rats
Source: Biomed Res Int. 2021 Feb 20;2021:6633825. doi: 10.1155/2021/6633825 (PMC7914094; doi:10.1155/2021/6633825)
Supplement: Supplementary Materials — Supplementary Figure 1: the systolic blood pressure (SBP), body weight, and representative flow cytometric analysis revealed the proportion of CD4+IL-17A+ (Th17) and CD4+FoxP3+ (Treg) cells by the gating strategy of flow cytometry in WKY and SHR. (a) SBP was measured using the tail-cuff method. SBP of SHR significantly increased by aging compared with that of WKY. (b) The body weights of the WKY and SHR had been monitored weekly for 13 weeks from 4 to 16 weeks. Data are the mean ± SEM of 6 independent experiments. Most SEMs were too small to be seen out of symbols (∗∗P < 0.01 vs. WKY by repeated measures ANOVA). Representative flow cytometric analysis revealed the proportion of CD4+IL-17A+ (Th17) cells and CD4+FoxP3+ (Treg) cells of the (c) PBMCs and (d) spleen in adult WKY or SHR. Supplementary Figure 2: protection of hypertension development in adult WKY from transfer of Th17 cells of adult SHR. (a) Schematic illustration shows that Th17 cells from adult donor WKY or SHR or the vehicle were transferred into adult recipient WKY. (b) SBP was measured in adult recipient WKY for 4 days using the tail-cuff method. SBP was not significantly increased in adult recipient WKY when either vehicle or transfer of Th17 cells from adult donor WKY or SHR was used. Data are the mean ± SEM of 4 independent experiments. Supplementary Figure 3: isolation strategy of Th17 cells from PBMCs of adult SHR. Single-cell suspension of PBMCs from adult SHR was analyzed based on forward scatter (FSC) and side scatter (SSC). Th cells were gated based on the expression of CD3+CD4+ and then further gated to Th17 cells by the expression of IL-17A. Supplementary Figure 4: SBP measurement and T cell profiles of juvenile recipient WKY and SHR in the PBMCs, spleen, and kidney for 4 days after transferring Th cells or Th17 cells from adult donor SHR. In the recipient WKY group, (a) there was no significant change in blood pressure when either vehicle or Th cells or Th17 cells of adult SHR were transfe [file 6633825.f1.pptx]

## Slide 1
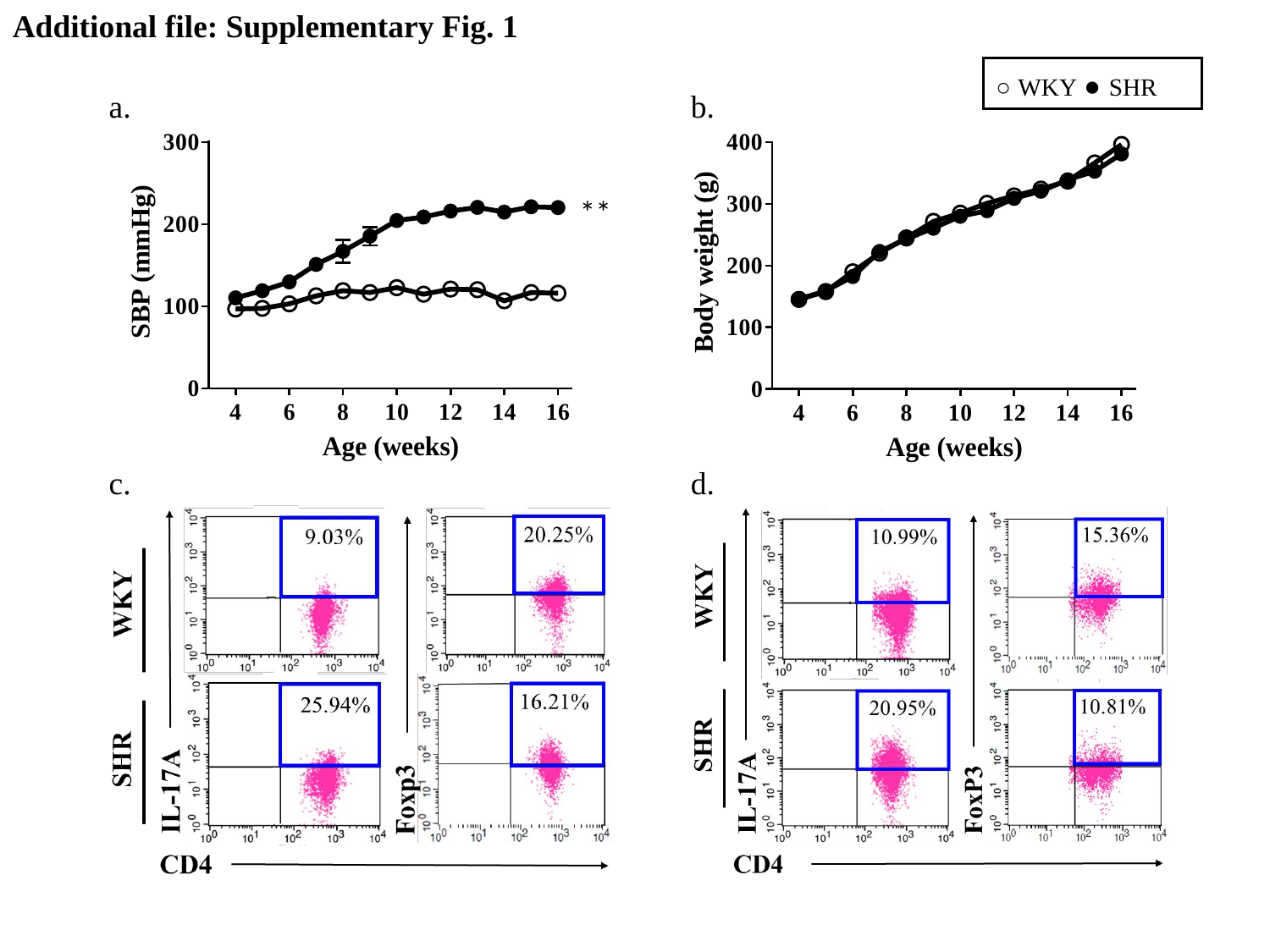

Additional file: Supplementary Fig. 1
○ WKY ● SHR
a.
b.
**
c.
d.

## Slide 2
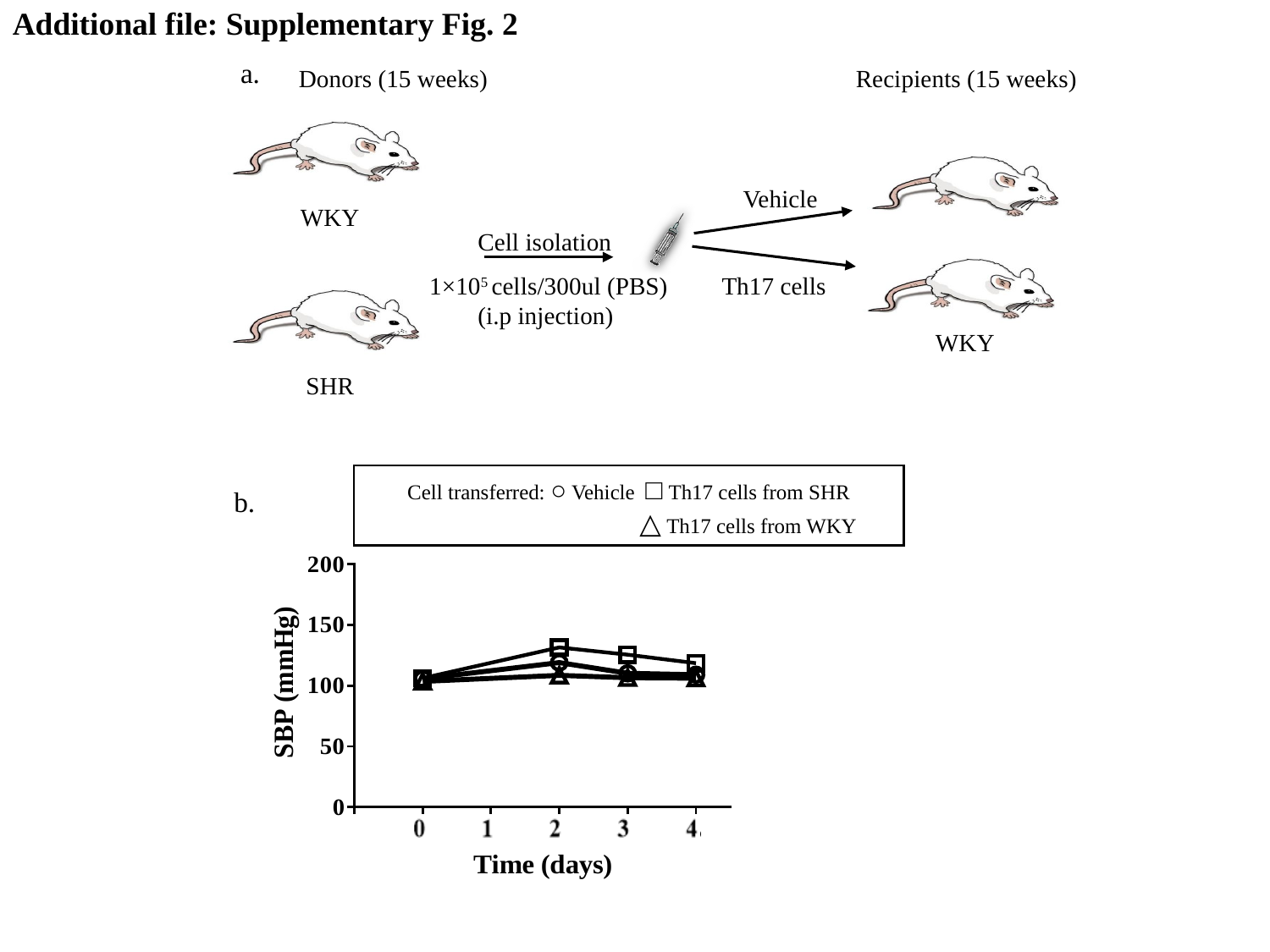

Additional file: Supplementary Fig. 2
a.
Recipients (15 weeks)
Donors (15 weeks)
Vehicle
WKY
Cell isolation
1×10^5^ cells/300ul (PBS)
(i.p injection)
Th17 cells
WKY
SHR
Cell transferred: ○ Vehicle □ Th17 cells from SHR
 △ Th17 cells from WKY
b.

## Slide 3
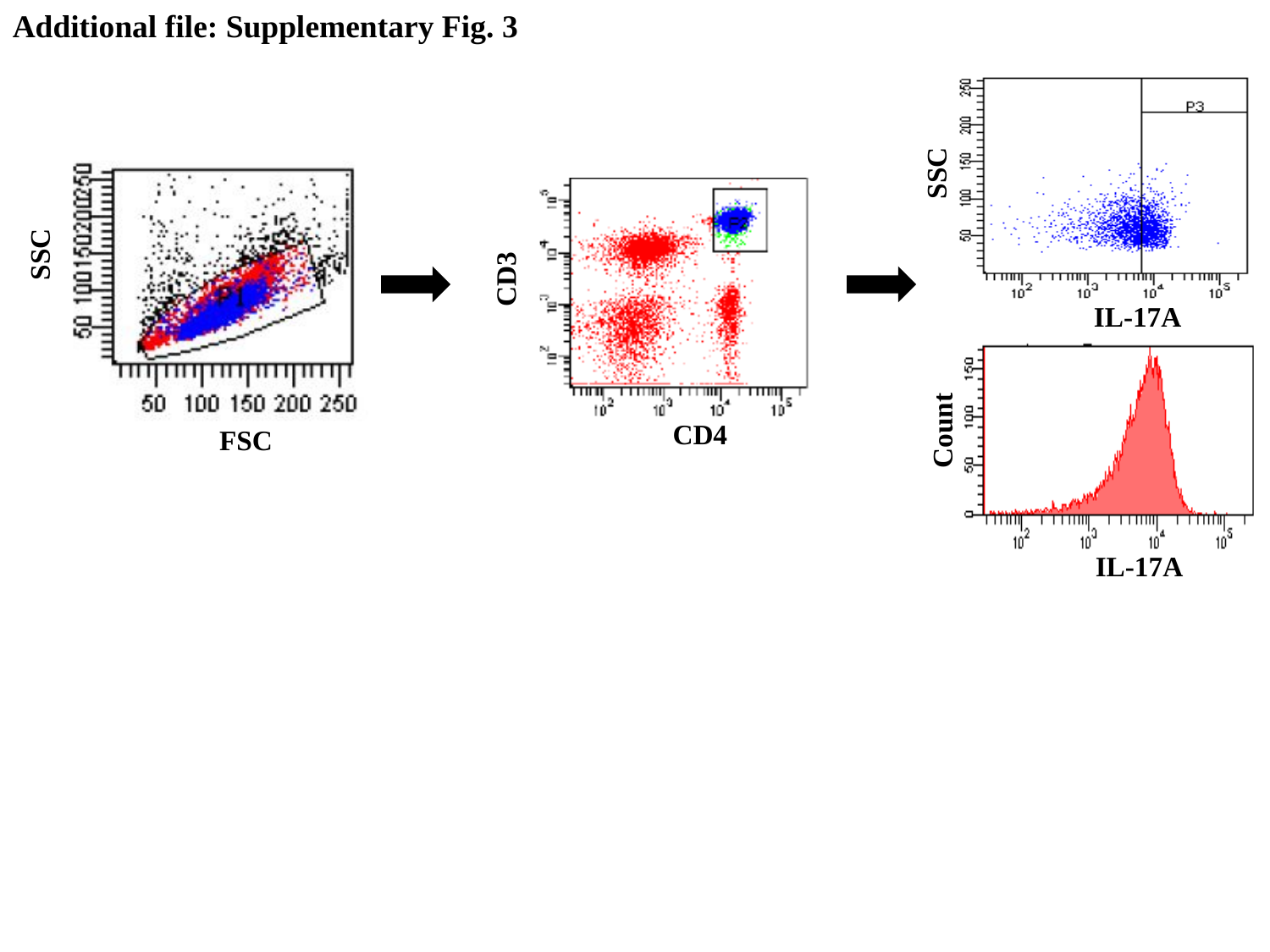

Additional file: Supplementary Fig. 3
SSC
SSC
CD3
IL-17A
Count
CD4
FSC
IL-17A

## Slide 4
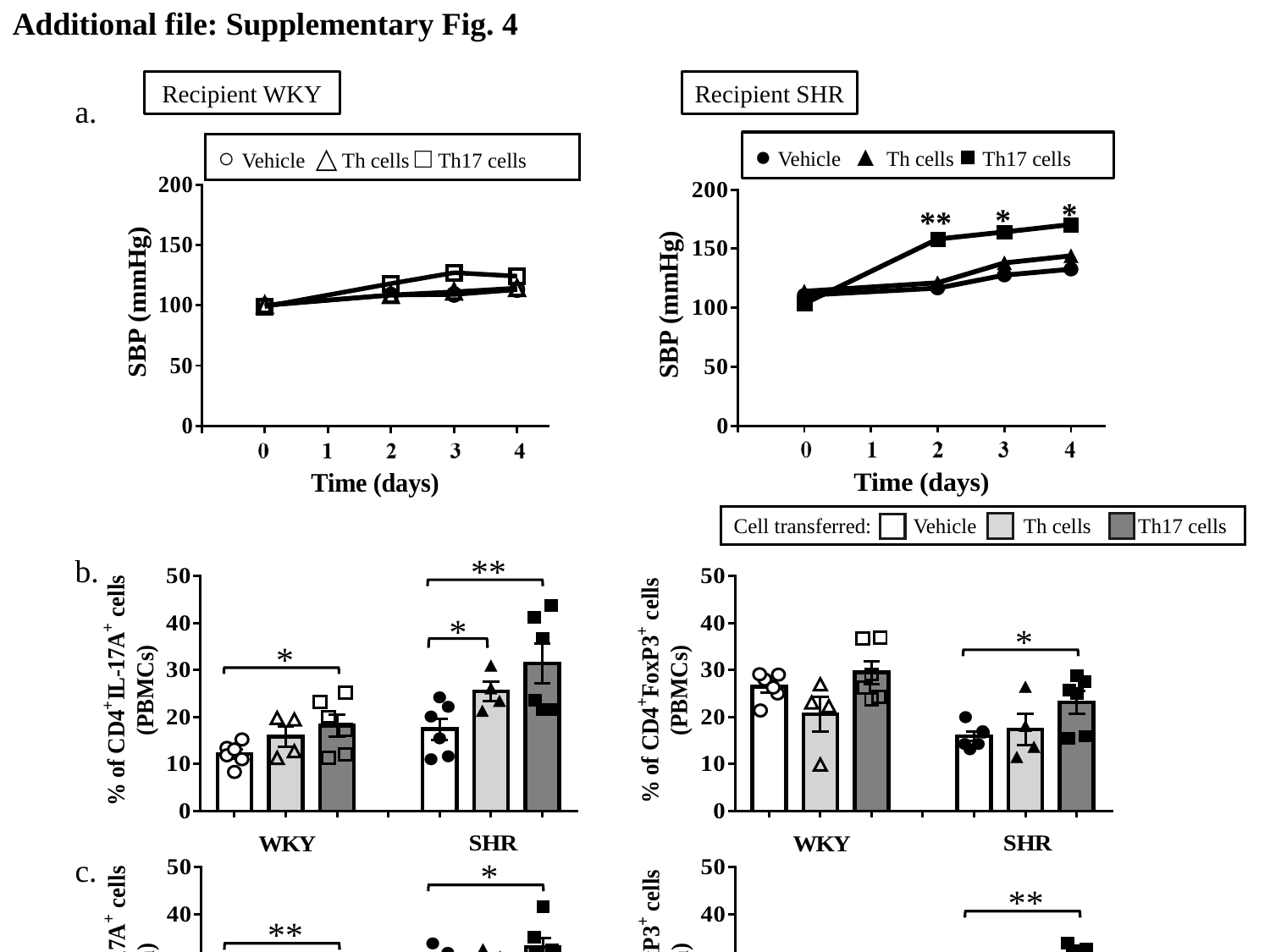

Additional file: Supplementary Fig. 4
Recipient WKY
Recipient SHR
a.
● Vehicle ▲ Th cells ■ Th17 cells
○ Vehicle △ Th cells □ Th17 cells
*
*
**
Cell transferred: Vehicle Th cells Th17 cells
**
b.
*
*
*
c.
*
**
**
*
d.

## Slide 5
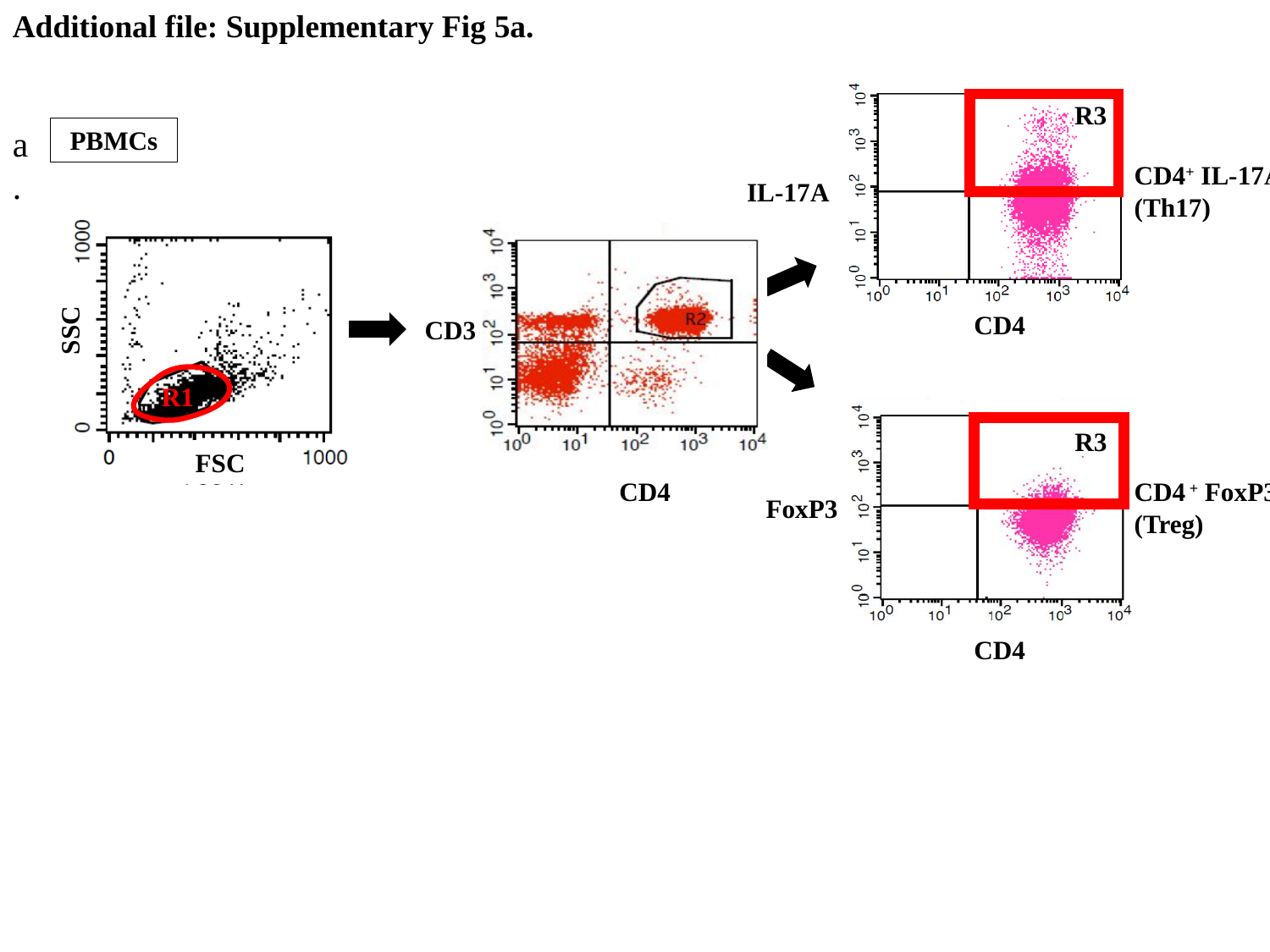

Additional file: Supplementary Fig 5a.
R3
a.
PBMCs
CD4+ IL-17A+
(Th17)
IL-17A
CD4
CD3
SSC
R1
R3
FSC
CD4
CD4 + FoxP3 +
(Treg)
FoxP3
CD4

## Slide 6
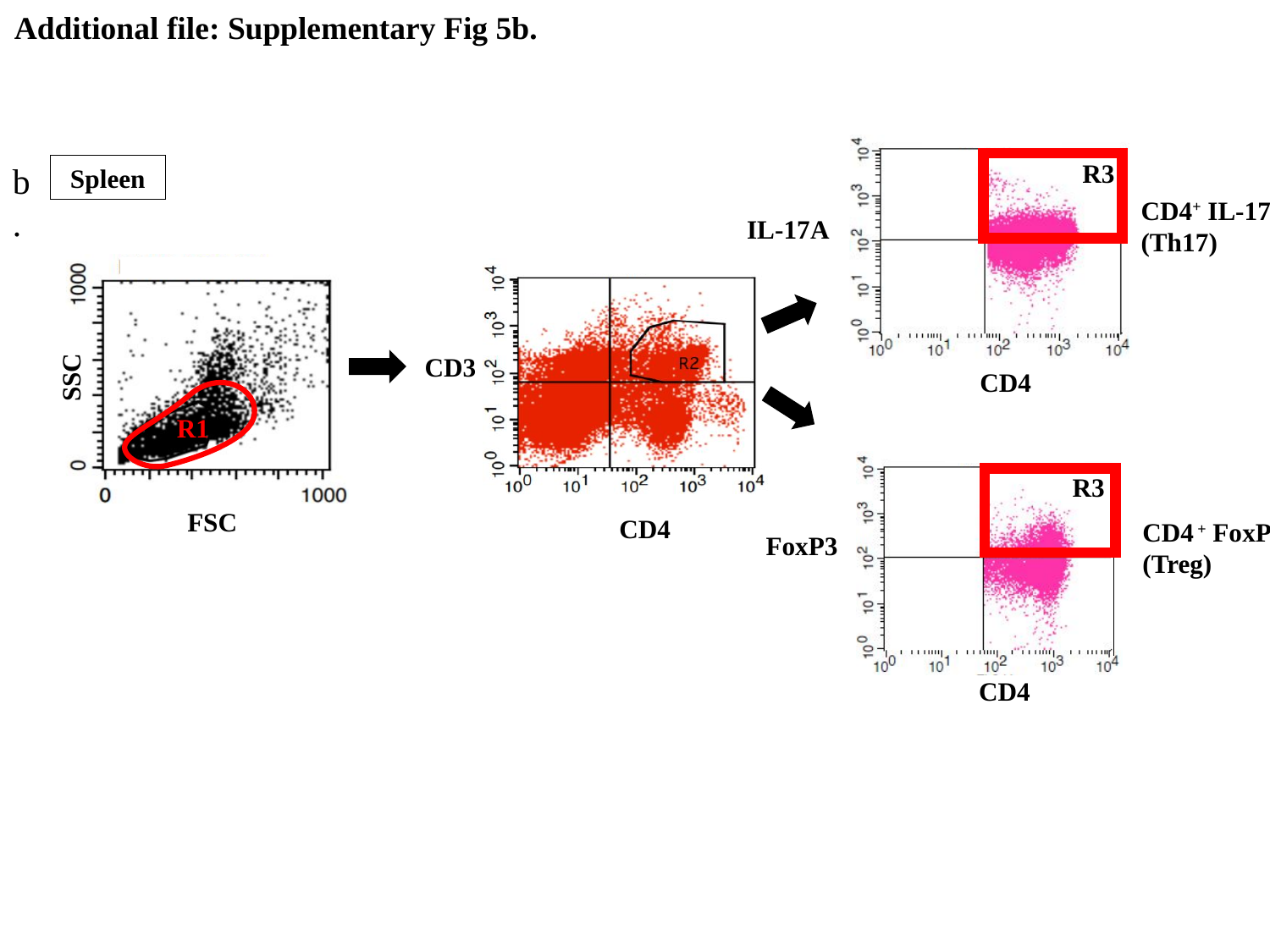

Additional file: Supplementary Fig 5b.
R3
b.
Spleen
CD4+ IL-17A+
(Th17)
IL-17A
CD3
SSC
CD4
R1
R3
FSC
CD4
CD4 + FoxP3 +
(Treg)
FoxP3
CD4

## Slide 7
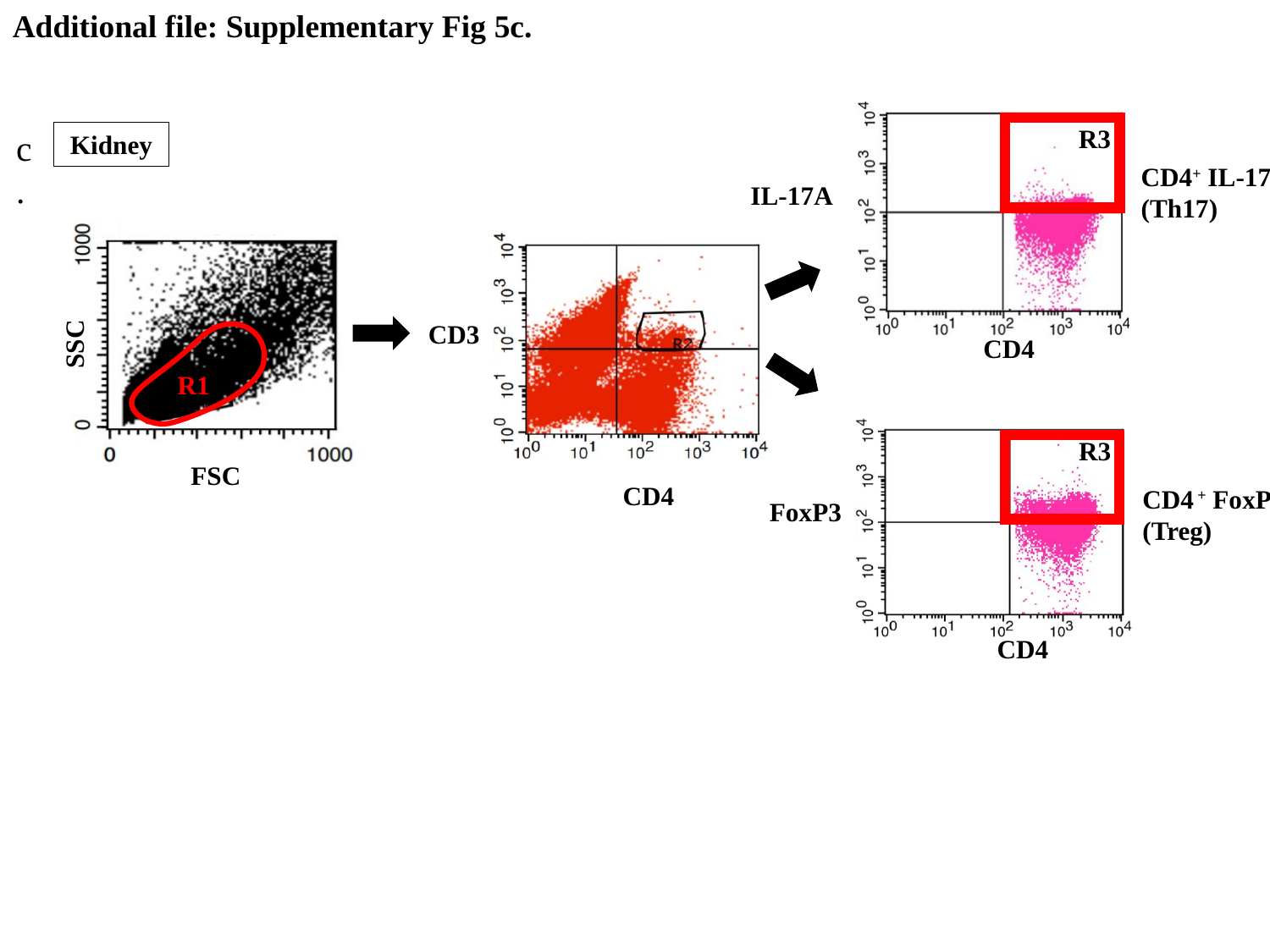

Additional file: Supplementary Fig 5c.
R3
c.
Kidney
CD4+ IL-17A+
(Th17)
IL-17A
CD3
SSC
CD4
R1
R3
FSC
CD4
CD4 + FoxP3 +
(Treg)
FoxP3
CD4
